# Up-regulation of p16 by miR-877-3p inhibits proliferation of bladder cancer

**DOI:** 10.18632/oncotarget.10575

**Published:** 2016-07-13

**Authors:** Shiqi Li, Yi Zhu, Zhen Liang, Xiao Wang, Shuai Meng, Xin Xu, Xianglai Xu, Jian Wu, Alin Ji, Zhenghui Hu, Yiwei Lin, Hong Chen, Yeqing Mao, Wei Wang, Xiangyi Zheng, Ben Liu, Liping Xie

**Affiliations:** ^1^ Department of Urology, The First Affiliated Hospital, School of Medicine, Zhejiang University, Hangzhou, 310003, Zhejiang, PR China

**Keywords:** bladder cancer, microRNA-877-3p, p16, RNA activation

## Abstract

Despite the recent studies which have shown that microRNA (miRNA) negatively regulates gene expression by silencing the expression of target genes, here we reported the new evidence of microRNA-mediated gene activation by targeting specific promoter sites. We identified a miR-877-3p binding site on the promoter site of tumor suppressor gene p16 which alters frequently in bladder cancer. Enforced expression of miR-877-3p could increase the expression of p16, which inhibit the proliferation and tumorigenicity of bladder cancer through cell cycle G1-phase arrest. Further evidences confirmed that the correlation between p16 activation and miR-877-3p was due to the direct binding. These findings demonstrate the anti-tumor function of miR-877-3p in bladder cancer cells and reveal a new pattern of miRNA involved gene regulation.

## INTRODUCTION

Urinary bladder cancer is the most common urogenital malignant tumor which ranks as the sixth most common cancer worldwide [1]. Despite the significant advances in therapies, high rate of recurrence and progression of noninvasive UBC and poor outcomes of the muscle invasive UBC impel us to seek for more effective treatments.

MicroRNAs (miRNAs) are small endogenous, non-coding RNAs with approximately 22 nucleotides that induce post-transcriptional gene regulation [2]. Aberrant expressions of miRNAs have been verified in numerous of tumors and evidence indicate that miRNAs are involved in tumor pathogenesis, including tumor proliferation, apoptosis, differentiation, invasion and metastasis [3, 4]. Despite the typical gene repression regulation of miRNAs through binding to complementary sequences in the 3′ untranslated regions (3′-UTR) of target mRNAs, studies have demonstrated that miRNAs are able to target 5′-UTR, open reading frame and promoter region of specific genes as well [2, 5, 6]. Similar to the RNA activation (RNAa), which are described as synthetic double-stranded RNAs induced gene expression activation, miRNAs are capable of up-regulating the expressions of specific genes by targeting promoters [7, 8].

P16 (CDKN2A) is known to be an important tumor suppressor gene functioning as cell cycle regulator [9, 10]. Aberrant expressions of p16 which occur as frequently mutated or deleted are found in a wide variety of tumors [9, 11]. Thus restoration of the function of p16 is an appropriate strategy to inhibit the proliferation of cancer cells.

In our study, we scanned the p16 promoter sequences and identified miR-877-3p as the gene activator. Overexpression of miR-877-3p could suppress the bladder cancer growth via activating the expression of p16 gene.

## RESULTS

### MicroRNA predicted to target p16 promotor

**Figure 1:**
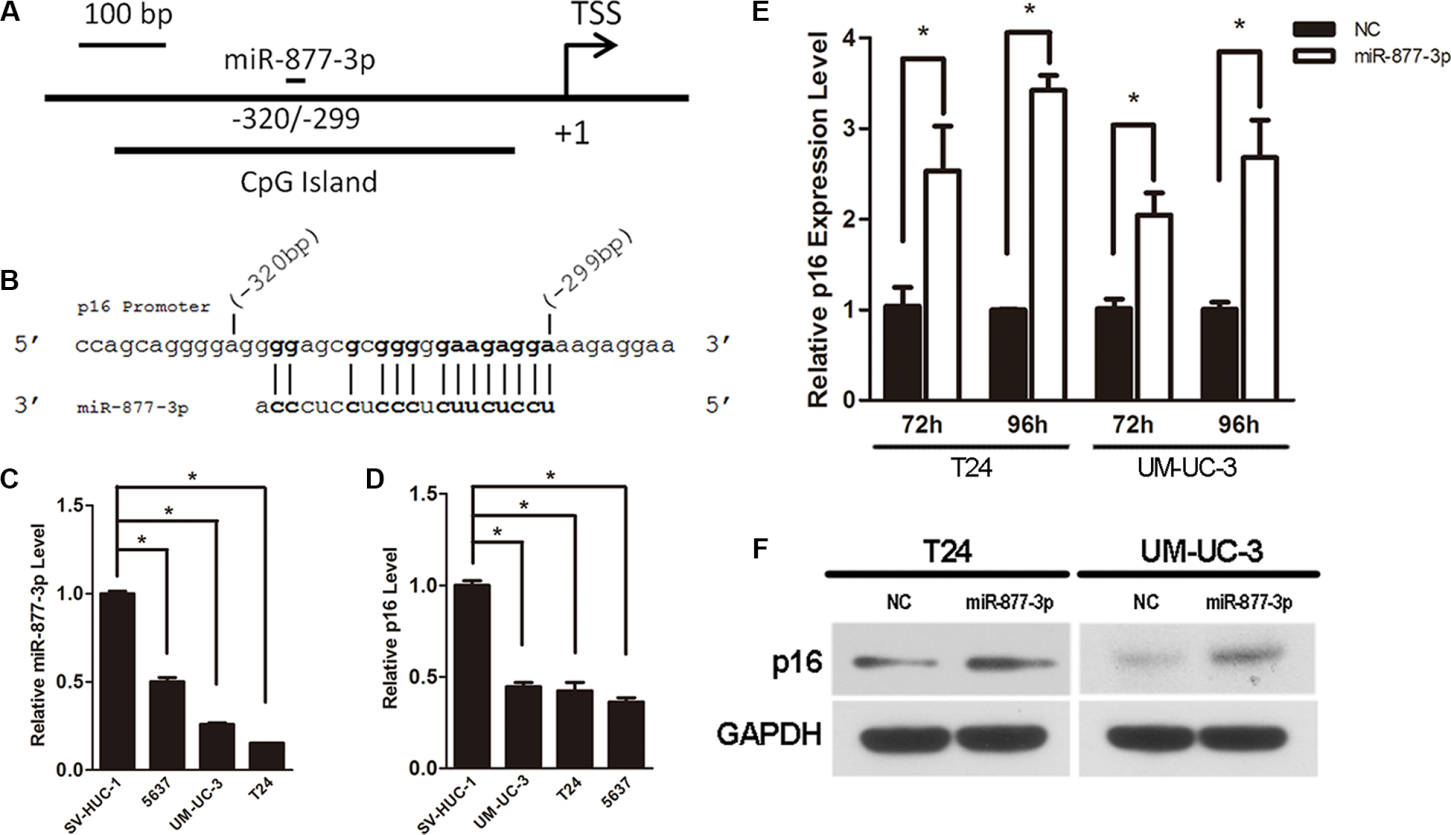
Activation of p16 expression by miR-877-3p in bladder cancer cells Expression levels for miR-877-3p and p16 by real-time PCR analysis were normalized with U6 and GAPDH, respectively. (**A**) Schematic representation of p16 promoter and miR- 877- 3p target location relative to TSS. (**B**) MiR-877-3p sequence and the complementary sequence of p16 promoter. (**C** and **D**) The miR-877- 3p and p16 expression levels in bladder cancer cell lines (5637, UM-UC-3 and T24) were measured and compared with non- tumor urothelial cell line SV-HUC-1(*P < 0.05). (**E**) mRNA levels of p16 in T24 and UM-UC-3 cells after 72 h or 96 h transfection with 50 nM of miR- 877- 3p or NC (*P < 0.05). (**F**) Western blot analysis was performed to detect the protein expression of p16 in T24 and UM-UC-3 cells 72 h following treatment with miR-877-3p or NC. GAPDH was used as inter control.

We scanned 1.5 kb of p16 promoter to search for probable complementary site of known human miRNAs. Several miRNAs were identified by online prediction tool. After detecting the expression pattern of these miRNAs in bladder cancer cells, we finally chose miR-877-3p for further experiments (Figure 1A and 1B).

### Expression patterns of miR-877-3p and p16 in bladder cancer cells

In order to verify the expression of miR-877- 3p in human bladder cancer, real-time RT-PCR was performed to quantify and analyze the expression levels of miR- 877- 3p in three kinds of bladder cancer cell lines (T24, UM-UC-3 and 5637 cell lines) versus SV-HUC-1 cell (a normal transitional epithelial cell line). The results of real-time RT-PCR revealed that compared with SV-HUC-1 cell line, expressions of miR-877-3p in three bladder cancer cells were down-regulated with more than 50% reduction (Figure 1C), which indicated that miR- 877- 3p might be a tumor suppressor in bladder cancer.

We further measured the expression of p16 in three bladder cancer cells and in SV-HUC-1 cell line with real-time RT-PCR. It turned out that all three bladder cancer cell lines exhibited a lower expression level of p16 compared with SV-HUC-1 cell line (Figure 1D).

### Overexpression of miR-877-3p activates p16 expression

To determine whether miR-877-3p could induce the expression of p16 in bladder cancer cells, synthetic miRNAs mimics of miR-877-3p or NC were transfected into T24 and UM-UC-3 cells. Real-time PCR demonstrated that compared with the negative control, the mRNA levels of p16 in T24 cells after 72 h or 96 h transfection were increased to 2.7– and 3.7– fold, respectively (Figure 1E). The results of UM-UC-3 cells showed the consistent expression pattern. 2.1- and 2.4– fold increasing were observed after 72 h or 96 h transfection, respectively (Figure 1E).

Western blotting was performed to further verify the activation of p16 by miR-877-3p in protein levels. It turned out that the protein levels of p16 in both T24 and UM-UC-3 cells were raised after transfected with miR- 877-3p mimics for 72 h (Figure 1F).

The above results manifested that overexpression of miR-877-3p could active the p16 expression in bladder cancer cells on both mRNA and protein levels.

### miR-877-3p activates the expression of p16 through binding to p16 promoter

**Figure 2:**
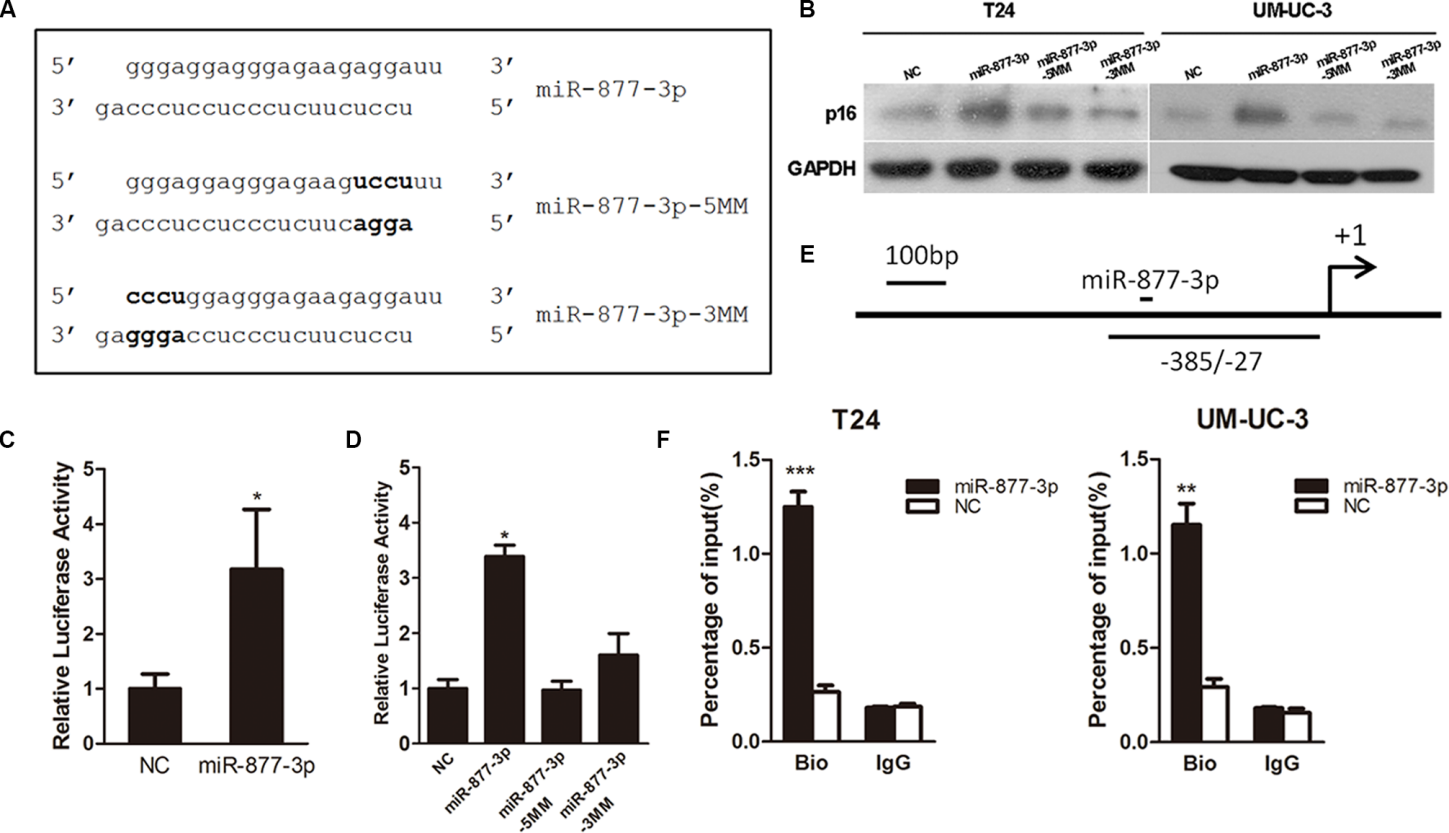
miR-877-3p interacts directly with p16 promoter (**A**) The original sequence and mutant sequence of miR-877-3p. (**B**) Western blot analysis of p16 expressions in T24 and UM-UC-3 cells treated with miR-877-3p and its mutants. (**C** and **D**) T24 cells were co-transfected with 50 nM of NC or miR-877-3p or its mutants and 500 ng pGL-3 Basic Vector carrying the target region and 25 ng pRL. The relative firefly luciferase activity normalized with Renilla luciferase was measured 72 h after transfection (*P < 0.05). (**E**) Schematic illustration of the location of primers capable of amplifying the miR-877-3p target sequence of p16 promoter. (**F**) T24 and UM-UC-3 cells were transfected with 50 nM of 3′-biotinylated miR-877-3p or NC for 72 h. The anti-biotin antibody was used to pull down miR-877-3p linked DNA. The DNA was amplified by real-time PCR with appropriate primer and normalized to input levels. IgG was used as a negative control. ChIP assay confirmed miR-877-3p enrichment at target region of p16 promoter in both T24 and UM-UC-3 cells (**P < 0.01 and ***P < 0.001).

A luciferase reporter assay was performed to testify the correlation between miR-877-3p and p16 promoter region. A PGL-3 Basic Vector containing a 1.5- kb promoter sequence of p16 which included the target region of miR-877-3p was constructed and a pRL (Ranilla Luciferase Control Reporter Vector) was used as an internal control. These reporter vectors were co- transfected into T24 cells with miR-877-3p or NC which served as a negative control. Overexpression of miR-877-3p showed increased luciferase activity compared with the negative control (Figure 2A and 2C), which demonstrated that the enhanced activity of p16 promoter was caused by miR-877-3p. In addition, two miR-877-3p mutants were synthesized to create mismatches with the target region. Each of the mutants contained 4 bases mutation of either 5′- or 3′- end of miR- 877-3p. It turned out with no surprise that both of the mutants failed to increased luciferase activity (Figure 2A and 2D). Meanwhile, western blotting confirmed that the miR-877-3p mutants could not increase the expression of p16, which indicated that the complete sequence was required for the activation of p16 by miR- 877-3p (Figure 2B).

We further tried to figure out whether the enhanced activity of p16 promoter caused by miR-877-3p was through direct binding. We conducted the ChIP assay with biotin covalently linked to the 3′- end of miR-877- 3p and NC antisense. 72 h after transfected with either miR- 877- 3p or NC, a well-characterized biotin antibody was used to pull down the target promoter DNA. Real-time PCR was performed to verify DNA enrichment with specific primers. We found that in both T24 and UM-UC-3 cells, biotin labeled miR-877-3p was able to pull down promoter DNA more efficiently (Figure 2E and 2F).

Taken together, we considered that miR-877-3p was able to activate the expression of p16 via directly binding to p16 promoter.

### miR-877-3p inhibits the proliferation and tumorigenicity of bladder cancer cells *in vitro* and *in vivo* and triggers G1-phase arrest by inhibiting downstream genes of p16

**Figure 3:**
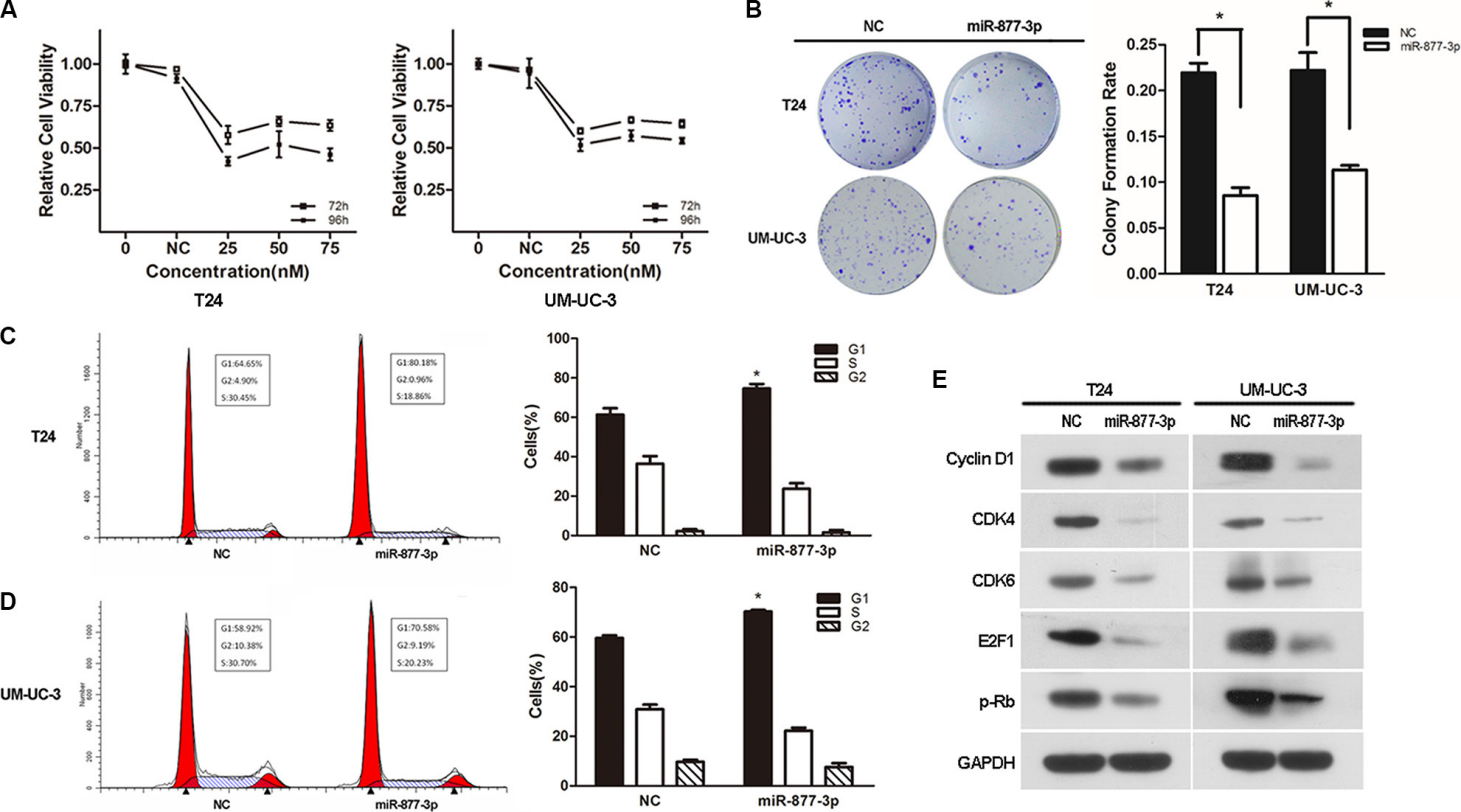
Over-expression of miR-877-3p inhibits bladder cancer proliferation and reduces the expression of the downstream genes of p16 (**A**) Cell growth/cell viability assay. The relative cell viability of the miR-877-3p transfected groups in T24 and UM-UC-3 cells were lower than that of transfected with NC (cell viability of 0 nM was regarded as 1.0), respectively (*P < 0.05). In both cell lines, treated with 25 nM of miR-877-3p for 96 h showed the best inhibition ability. (**B**) Colony formation assay (Representative wells were presented). The colony formation rate was lower in miR-877-3p treated groups compared with NC groups, (*P < 0.05). (**C** and **D**) Cell cycle distribution in T24 and UM-UC-3 cell lines. Over-expression of miR-877-3p caused significant G1-phase arrest in both of the cell lines (Representative histograms are shown above. The indicated percentages are the average of triplicate experiments) (*P < 0.05). (**E**) Western blot analysis of downstream genes of p16 in T24 and UM-UC-3 cell lines. MiR-877-3p induced change of downstream genes of p16, which were also G1/S transition regulators (CDK4, CDK6, cyclinD1, E2F1 and p-RB). GAPDH was served as a normalizer.

As we mentioned before that miR-877-3p might act as a tumor suppressor, we performed the gain-of-function experiments to detect its effect on bladder cancer. CCK- 8 and colony formation assays showed that compared with NC-transfected cells, overexpression of miR-877-3p could suppress the growth of bladder cancer cells. Treated for 96 h at the contraction of 25 nM, cell viability of T24 and UM-UC-3 cell was reduced by 58% and 49%, respectively (Figure 3A). The colony formation assay displayed the similar results that the colony formation rate was decreased significantly in both of the bladder cancer cell lines (Figure 3B).

**Figure 4:**
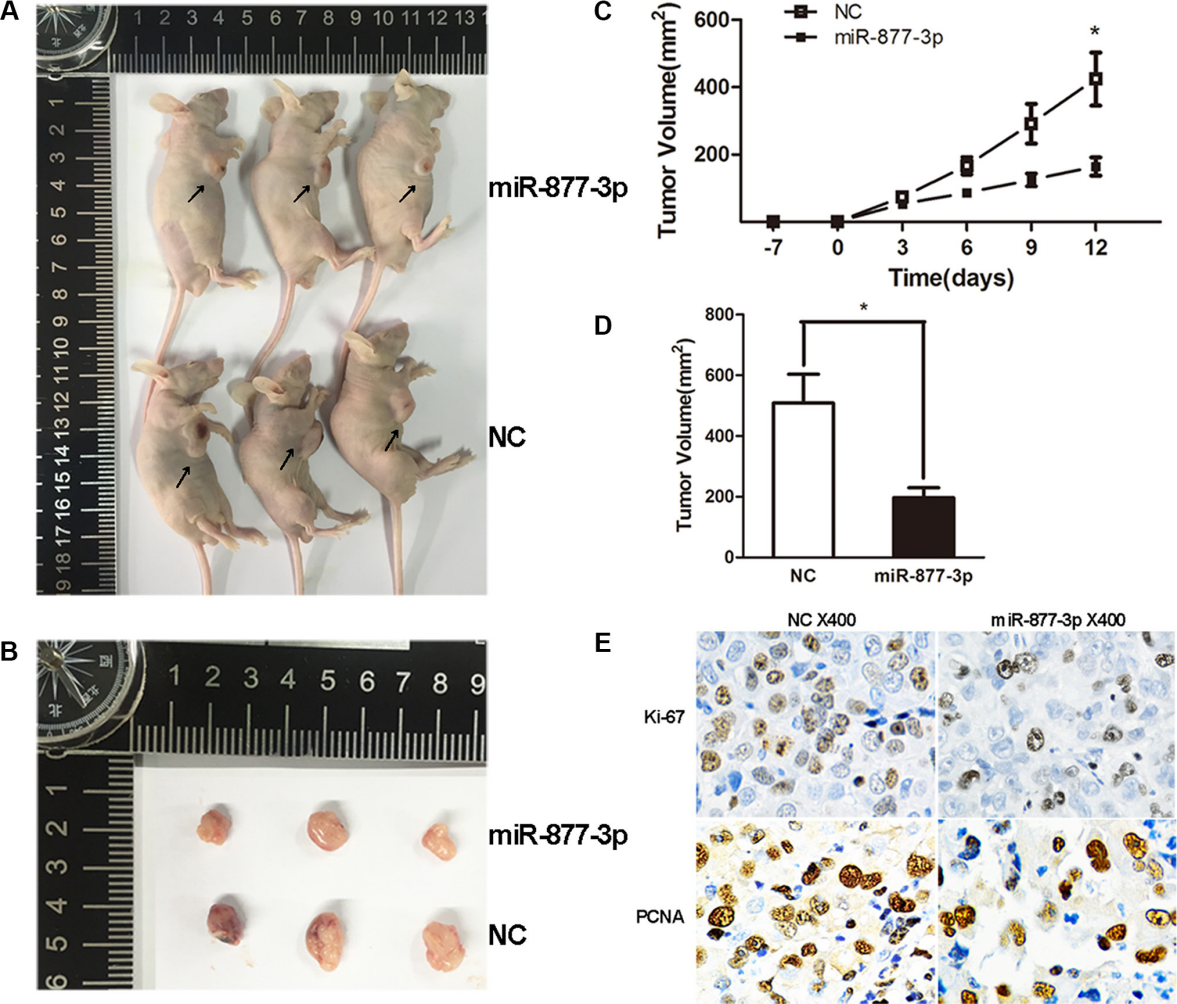
Tumor xenograft model (**A–D**) The tumor volumes and the growth curves suggested that tumors treated with miR-877- 3p displayed a slower growth pattern compared with non-miR-877-3p treated group. Error bars represent the S.D. from three nude mice (*P < 0.05). (**E**) IHC staining showed decreased Ki-67 and PCNA expressions in miR-877-3p treated tumor tissues.

For further certification, miR-877-3p was transduced into tumor tissues of T24 tumor xenograft BALB/c-nude mice. We observed tumor growth retardation in the mice treated with miR-877-3p. (Figure 4A–4D) In addition, the IHC staining indicated decreased expression pattern of Ki-67 and PCNA in miR-877-3p-overexpressing tumor tissues (Figure 4E), which confirmed that miR-877-3p negatively regulate the growth of bladder cancer.

Moreover, flow cytometry was performed to reveal the underlying mechanism of miR-877-3p-mediated growth suppression. Both T24 and UM-UC-3 cells transfected with miR-877-3p exhibited increased percentage of cell distribution in the G1/G0 phase, along with the decreased percentage in S phase (Figure 3C and 3D), which proved that miR-877-3p could inhibit the proliferation of bladder cancer cells via G1-phase arrest. Consistently, Western Blotting showed decreased expression of the downstream effector proteins of p16 which involved in the G1/S transition regulation, including CDK4/6 and Cyclin D1. Also, expression E2F1 and p-Rb which are participating in cell cycle regulation were observed to be decreased (Figure 3E).

### miR-877-3p induces cell cycle arrest mainly by activating the expression of p16

**Figure 5:**
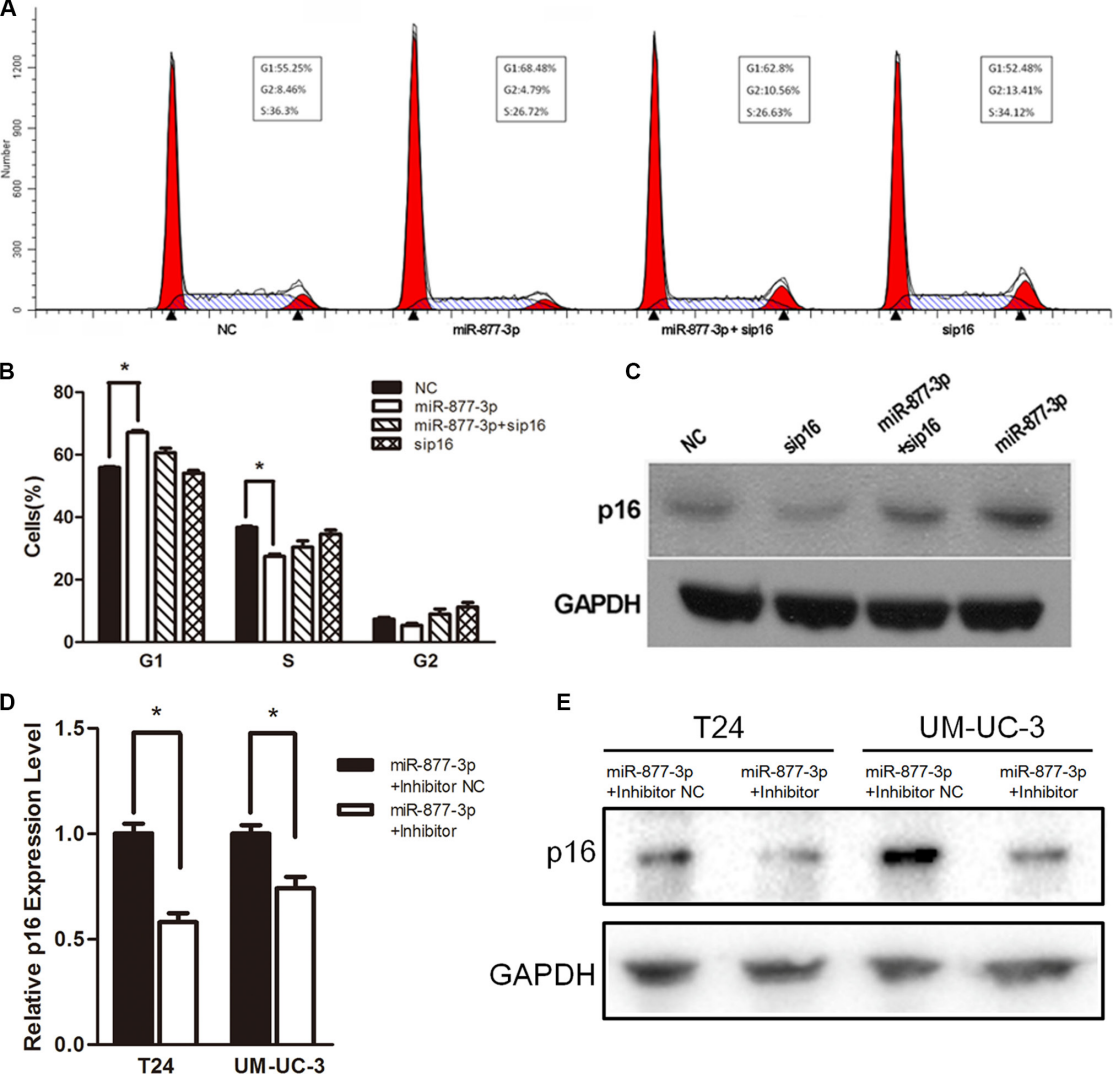
miR-877-3p induces cell cycle arrest mainly by upregulating p16 T24 cells were transfected with 50 nM of miR- 877- 3p, siP16 or NC for 72 h. (**A** and **B**) Reduced expression of p16 partly abrogated miR-877-3p-medated cell cycle arrest (*P < 0.05). (**C**) Expression of p16 protein was detected by Western Blot analysis. GAPDH was used as a loading control. (**D** and **E**) T24 and UM-UC-3 cells were co-transfected with 50 nM of miR-877-3p and 100 nM of either miR-877-3p inhibitor or inhibitor NC for 72 h. Relative p16 mRNA levels were analyzed by real-time PCR (*P < 0.05). And the protein levels of p16 were accessed by Western Blot.

We have already proved that miR-877-3p could active the expression of p16 as well as cause cell cycle arrest in bladder cancer cells. Next, we were wondering whether the cell cycle arrest in bladder cancer cells was depending on the increased expression of p16. Therefore RNA interference (siP16) was used to knockdown the expression of p16 in T24 cells (Supplementary Figure S3) and Western blotting demonstrated that siP16 could abrogate the increased expression of p16 by miR-877- 3p (Figure 5C). The results of flow cytometry showed that reduced expression of p16 significantly abolished the miR-877-3p-mediated cell cycle arrest (Figure 5A and 5B), which implied that the activation p16 was mainly responsible for the cell cycle arrest in bladder cancer cells.

In addition, knock-down of miR-877-3p by co- transfection with miR-877-3p mimic and inhibitor could decrease the expression of p16 on both mRNA and protein levels (Figure 5D and 5E), which further proved that the increased expression of p16 was specific to the sequence of miR-877-3p.

## DISCUSSION

In the present study, we verified the low expression pattern of p16 in bladder cancer cells and searched for probable microRNAs targeting p16 promoter. It turned out that miR-877-3p which targeted the −320~–299 site of the p16 promoter could increase the expression of p16 in bladder cancer cells on both mRNA and protein levels, which was because of the direct binding between miR-877-3p and p16 promoter. Gain-of-function study showed that overexpression of miR-877-3p could inhibit the proliferation *in vitro* and *in vivo* via G1-phase arrest. To sum up, we demonstrated that miR-877-3p had an anti-tumor function in bladder cancer cells which was achieved mainly through activating the expression of p16.

In recent years, plenty of researches have shown that miRNAs play essential roles in bladder cancer progression as well as regulating signal pathways and turned out to be promising therapeutic targets [12–15]. Typically, miRNAs suppress the expression of target genes by partly binding to complementary sequences in the 3′-UTR of target mRNAs, which would induce the inhibition of translation and the degradation of target mRNAs [2, 16]. However, more evidences indicated that the miRNA mediated gene expression regulation had more possibilities and 5′-UTR, coding sequence or promoter of the genes could be the target region as well [5, 6]. Several tumor suppressor genes, including E-cadherin, IL-24, IL-32 and p21 have been reported to be activated by miRNAs through targeting specific sites in their promoters [17–19]. As in our study, miR-877-3p which was partly complementary to the promoter region of p16 could induce the expression of p16 after transfection for 72 h and the activation effect showed to be even stronger with a longer treatment time of 96 h.

Our study conducted Luciferase assays and Chip assay to validate the direct correlation between miR- 877- 3p and p16 promoter. In the Luciferase assays, we observed relatively higher activity of p16 promoter after miR-877- 3p treatment, which proved the induction of p16 promoter activity was specific to the sequence of miR-877-3p. Moreover, the biotin covalently linked miR-877-3p showed better performance of pulling down promoter DNA in the chip assay, which provided stronger evidence of miR-877- 3p directly binding to p16 promoter. However, the exact mechanism of miRNA mediated gene activation still remains elusive. Variations of target locations, thermodynamic properties and chromatin/DNA accessibility may all have influences on the susceptibility of gene activation [20]. It has been noted that DNA methylation might affect gene expression and involved in the RNA-mediated gene regulation [21, 22]. With the assistance of the CpG Island Searcher program (http://www.urogene.org/methprimer/), we identified that the target site of p16 promoter was in the CpG Island near the TSS, which meant that it might be hypermethylated. In order to figure out whether DNA methylation might take part in the activation of p16, we treated T24 cells with 5-Aza, a methyltransferase inhibitor. The result showed that there was no significant change in the expression of p16 (Supplementary Figure S1). In addition, after transfected with miR-877-3p in T24 cells, DNA methyltrans-ferases, including Dnmt1, Dnmt3a and Dnmt3b displayed unaffected, either (Supplementary Figure S2). These findings might give us a hint that DNA methylation might not be involved in miR-877-3p mediated p16 activation. Similarly, reports have pointed out that DNA methylation might not be necessarily required in RNA-mediated transcriptional regulation [7, 8, 23].

pl6 (also known as CDKN2) is a tumor suppressor gene which decelerates cell progression from G1 phase to S phase by inhibiting cyclin dependent kinases such as CDK4 and CDK6 [9, 24]. Mutations in the p16 gene are related to increased risk of a wide range of cancers, including pancreatic adenocarcinoma, lung cancer and other tumors [11, 25, 26]. Restoring p16 expression could suppress the growth of various tumor types and has been regarded as a promising therapeutic target [27–29]. Alteration of the p16 and p15 genes which occurs at 9p21 appears to be a common event in bladder cancer and p16 has been proposed to be the major deletion target. The frequent inactivation of p16 has been shown to be associated with the progression of bladder cancer to a more malignant phenotype [30–32]. Meanwhile, transduction of p16 antitumor peptide displayed inhibition of bladder cancer in mouse model, which indicated the restoration of p16 to be a probable treatment of bladder cancer [33]. Our data suggested that the increased expression of p16 induced by miR-877-3p could inhibit the growth of bladder cancer *in vivo* and *in vitro* through G1 phase arrest. The decreased expression of CDK4, CDK6 which were directly inhibited by p16, along with the down-regulated expression of Cyclin D1, E2F1 and pRb confirmed the anti-tumor function of miR-877-3p was the due to p16 activation.

In summary, our study presented the anti-tumor function of miR-877-3p in bladder cancer cells through activating the expression of p16 by directly binding to the specific promoter site. It provided the evidence of miRNA-mediated activation which represented a novel miRNA involved gene regulation. The phenomenon that activation of tumor suppressor genes by miRNA may point to a new therapeutic strategy. Although further studies are required to identify the mechanism of miRNA activation, our research may lead to a new therapeutic approach for bladder cancer treatment.

## MATERIALS AND METHODS

### Target miRNA prediction

Online prediction tool RegRNA2.0 was used to detect the miRNA candidate complementary to p16 gene promoter. 1.5 kilobase of human p16 promoter sequence was derived from NCBI gene database (http://www.ncbi.nlm.nih.gov/), and human miRNA sequence was derived from miRBase (www.mirbase.org). We set the alignment score greater than 170 and free energy less than minus 25 as the prediction standard and all known miRNAs were scanned against the p16 promoter sequence. In addition, we verified that the candidate miR-877-3p did not possess putative target sites in 5′ or 3′ UTR of the p16 transcript.

### Oligonucleotide transfection

The miR-877-3p mimic (named as miR-877- 3p), miR-877-3p inhibitor, 3′-biotin covalently linked miR-877-3p, siRNA against p16 (named as sip16) and negative control duplex (named as NC and NC inhibitor) which lacks any significant homology to known human sequences were all chemically synthesized by GenePharma (Shanghai, China). Oligonucleotide transfection was performed using Lipofectamine 2000 reagents (Invitrogen, Carlsbad, CA, USA) according to the manufacturer's protocol. The sequences were listed in Table 1.

**Table 1:** The oligonucleotides used in this study

Name*	Sequence (5′– > 3′)
miR-877-3p (sense)	UCCUCUUCUCCCUCCUCCCAG
miR-877-3p inhibitor (sense)	CUGGGAGGAGGGAGAAGAGGA
sip16 (sense)	CACCAGAGGCAGUAACCAUTT
NC (sense)	ACUACUGAGUGACAGUAGA
NC inhibitor (sense)	CAGUACUUUUGUGUAGUACAA
U6-F	TGCGGGTGCTCGCTTCGGCAGC
p16-F	GGGTTTTCGTGGTTCACATCC
p16-R	CTAGACGCTGGCTCCTCAGTA
miR-877-3p-F	TCCTCTTCTCCCTCCTCCCAG
GAPDH-F	ACAACTTTGGTATCGTGGAAGG
GAPDH-R	GCCATCACGCCACAGTTTC
p16-Promoter-F	tcgaGCTAGCAAGCGCATGAACAGGAAGC
p16-Promoter-R	tcgaAAGCTTCCGGAGGGTCACCAAGAA
p16-320/−299-F	AACGGTCGCCAAGACAACCATTC
p16-320/−299-R	AACTAAACCGCTGCACGCCTCTG

* F, forward primer; R, reverse primer.

### Cell lines and cell culture

The human bladder cancer cell lines T24, UM-UC-3 and non-malignant cell line SV-HUC1 were purchased from Shanghai Institute of Cell Biology, Chinese Academy of Sciences. All the cells were cultured in RPMI 1640 medium supplemented with 10% fetal bovine serum, 50 μg/ml streptomycin and 50 U/ml penicillin under a humidified air atmosphere with 5% CO2 at 37°C.

### RNA isolation and qRT-PCR

Total RNA from cultured cells was extracted using RNAiso plus (Takara, Dalian, China) according to the manufacturer's instructions. One Step PrimeScript miRNA cDNA Synthesis Kit (Takara, Dalian, China) and PrimeScript RT reagent Kit (Takara, Dalian, China) were used respectively to perform RNA reverse transcription into miRNA cDNA and total cDNA. The miRNA and mRNA expression levels were detected by qPCR with the ABI 7500 FAST real-time PCR System (Applied Biosystems, Carlsbad, USA) using SYBR Green (Takara, Dalian, China) and were normalization with reference to expression of U6 and GAPDH, respectively. The 2-ΔΔCt method was used to calculate and quantify the expression levels of miR-877-3p and p16. All primers used were listed in Table 1.

### Cell proliferation assay

Approximately 5 × 10^3^ T24 or UM-UC-3 cells were plated in each 96-well plates and incubated overnight before transfection. The cells were transfected with miR-877-3p for 72-96 h with the concentration ranging from 25 nmol to 75 nmol. After 72 or 96 h, the medium were removed before adding the Cell Counting solution (WST- 8, Dojindo Laboratories, Tokyo, Japan) to each well. After incubation at 37°C for an hour, the absorbance of the solution was measured spectrophotometrically at 450 nm with MRX II absorbance reader (Dynex Technologies, Chantilly, VA, USA).

### *In vitro* colony formation assay

T24 and UM-UC-3 cells were transfected with 2′-O-Methyl modified duplexes and harvested for 24 h. Then the cells were seeded in new six-well plates at the density of 500 cells per well and cultured without interference for 10 days. The cells were stained with 0.1% crystal violet for observation and analysis. The colony formation rate was calculated with the formula colony formation rate = (number of colonies/number of seeded cells) ×100%.

### *In vivo* tumorigenicity assays

Male BALB/c-nude mice (4 weeks old), each weighing 18–20 g, were purchased from the Shanghai Experimental Animal Center, Chinese Academy of Sciences, Shanghai, China. T24 cells (1 × 10^6^ in 100 μl PBS) were injected subcutaneously into the right flank of each mouse. The mice were divided randomly into treatment and control groups when palpable tumors arose and were injected intratumorally with 30 μg of Lipofectamine 2000-encapsulated miR-877-3p or NC every 3 days for 12 days. Tumor growth was observed by caliper measurements of the two perpendicular diameters every 3 days, and the volume of the tumor was calculated with the formula V = (width^2^ × length × 0.5). All procedures were performed in accordance with the Regulations for the Administration of Affairs Concerning Experimental Animals (approved by the State Council of the People's Republic of China) and approved by Experimental Animal Ethics Committee of Zhejiang University.

### Immunohistochemistry (IHC) staining

After fixed with formalin, tumor tissues were dehydrated and embedded in paraffin. Before performing epitope retrieval, tissue sections were dewaxed and rehydrated. The slides were incubated with Ki-67 or PCNA primary antibodies overnight at 4°C and incubated with a HRP-conjugated secondary antibody for 50 minutes at room temperature. DAB was applied for color development and dark brown staining was considered as positive. Hematoxylin was used for counterstain. The images were taken under a microscope (Olympus, Tokyo, Japan) with appropriate magnification.

### Cell cycle analysis by flow cytometry

Cells were harvested 72 h after the RNA transfection. After washed with PBS, the cells were fixed in 75% ethanol overnight at −20°C. Then the cells were washed with PBS for twice and applied with DNA Prep Stain (Beckman Coulter, Fullerton, CA, USA) and RNase for 30 min. Next, the cell cycle analysis was performed by BD LSRII Flow Cytometry System with FACSDiva software (BD Bioscience, Franklin Lakes, USA). The data were analyzed by ModFit LT 3.2 software (Verity Software House, Topsham, USA) and the cell cycle distribution was described as the percentage of cells in G1, S, and G2 populations.

### Protein extraction and Western blotting

The cells were lysed in cell lysis buffer 72 h after RNA treatment and the total protein concentration of every lysate was calculated by the BCA Protein Assay kit (Pierce). Equal amounts of protein samples were loaded and separated by 10% SDS–polyacrylamide gels. After transferred to polyvinylidene difluoride membranes, the membranes were blocked for 1 h at room temperature and incubated with primary antibody for the following night at 4°C. After washing and incubated with the corresponding secondary antibody for 1 h at room temperature, the membranes were detected by Chemi-luminescence (ECL). The primary antibodies used were: anti-CDKN2A/p16INK4a (1:1000), anti-Cyclin D1 (1:2000), anti-CDK4 (1:2000), anti-CDK6 (1:2000), anti-E2F1 (1:2000), anti- phosphorylated Rb (pT356, 1:2000) and anti-GAPDH (1:5000). All the primary antibodies were obtained from abcam, Cambridge, UK.

### Luciferase assays

In order to construct the promoter reporter vector, a 1.5-kb promoter sequence of p16 which contained the target region of miR-877-3p was inserted into the pGL- 3 Basic Vector (Promega, Fitchburg, Wisconsin, USA) between Nhe I and Hind III sites. The insertion was verified by sequencing (Sangon, Shanghai, China). pRL (Ranilla Luciferase Control Reporter Vector) (Promega, Fitchburg, Wisconsin, USA) was used as an internal control. Mutations of 4 base pairs of miR-877-3p led to the mutant derivatives miR-205-5MM and miR-205-3MM. The primers (named as p16-Promoter-F, p16-Promoter-R) used were listed in Table 1.

T24 cells were plated on a 24-well plate for 24 h and then co-transfected with 50 nM of either miR-877-3p mimics or NC oligos and 500 ng pGL-3 Basic Vector and 25 ng pRL. For the mutant microRNA, 50 nM of either miR-205-5MM or miR-205-3MM was co-transfected with 500 ng PGL-3 Basic Vector and 25 ng pRL. The Luciferase Reporter Assay was performed according to the manufacturer's protocol 72 h after the co-transfection (Promega, Fitchburg, Wisconsin, USA).

### Chromatin immunoprecipitation (ChIP) assay

Cells were treated with 3′-biotin covalently linked miR-877-3p for 72 h and then collected for ChIP assay according to the manufacturer's instructions (Millipore, Billerica, Massachusetts, USA). Approximately 5.0 × 10^6^ cells were applied for one immunoprecipitation. RNase inhibitor (Sigma, St. Louis, Missouri, USA) was used to avoid the degradation of miRNA. After treated with 1% formaldehyde to crosslink for 10 min at 37°C, cells were washed with glycine and PBS and scraped from the dishes. Then the cells were resuspended with Nuclei Isolation Buffer and SCW Buffer and sonication (30 sets of 5-second pules at a power setting of 30%) was performed on wet ice. Chromations were applied with Magna ChIP protein A/G Magnetic Beads and immunoprecipitated with antibodies of biotin (Santa Cruz Biotechnology, Dallas, Texas, USA) and IgG (Millipore, Billerica, Massachusetts, USA) overnight at 4°C. After the antibody/antigen/DNA complex was collected and reverse cross-linked, DNA was purified and used as the template for real-time PCR to verify Chip DNA enrichment. Primers are available in Table 1.

### Statistical analysis

Statistical analysis was performed using SPSS V17.0 software. All data were derived from three different independent experiments and expressed as the mean ± standard deviation (SD). *T*-tests were applied for analyze and *P* < 0.05 was considered to be statistically significant.

## SUPPLEMENTARY MATERIALS


