# Correction: Up-regulation of p16 by miR-877-3p inhibits proliferation of bladder cancer

**DOI:** 10.18632/oncotarget.26624

**Published:** 2019-01-18

**Authors:** Shiqi Li, Yi Zhu, Zhen Liang, Xiao Wang, Shuai Meng, Xin Xu, Xianglai Xu, Jian Wu, Alin Ji, Zhenghui Hu, Yiwei Lin, Hong Chen, Yeqing Mao, Wei Wang, Xiangyi Zheng, Ben Liu, Liping Xie

**Affiliations:** ^1^ Department of Urology, The First Affiliated Hospital, School of Medicine, Zhejiang University, Hangzhou, 310003, Zhejiang, PR China

**This article has been corrected:** The correct Grant number information is given below:

**ACKNOWLEDGMENTS AND FUNDING**

This work was supported by the Public Welfare Technology Application Research project of Zhejiang Provincial Science, Technology Department of China (Grant No. 2014C33198), Zhejiang Provincial Natural Science Foundation of China (Grant No. LY16H160015), National Natural Science Foundation of China (Grant No.81372773, 81472375, 81402096, 81502215), scientific research foundation of the ministry of public health (WKJ2012-2-009), Zhejiang province key project of science and technology (2014C04008-2).

Original article: Oncotarget. 2016; 7:51773-51783. https://doi.org/10.18632/oncotarget.10575

