# Supplementary material for: Up-regulation of p16 by miR-877-3p inhibits proliferation of bladder cancer
Source: Oncotarget. 2016 Jul 13;7(32):51773–83. doi: 10.18632/oncotarget.10575 (PMC5239514; doi:10.18632/oncotarget.10575)
Supplement: Supplementary file 1 [file oncotarget-07-51773-s001.pdf]

Up-regulation of p16 by miR-877-3p inhibits proliferation of bladder cancer

Supplementary Materials

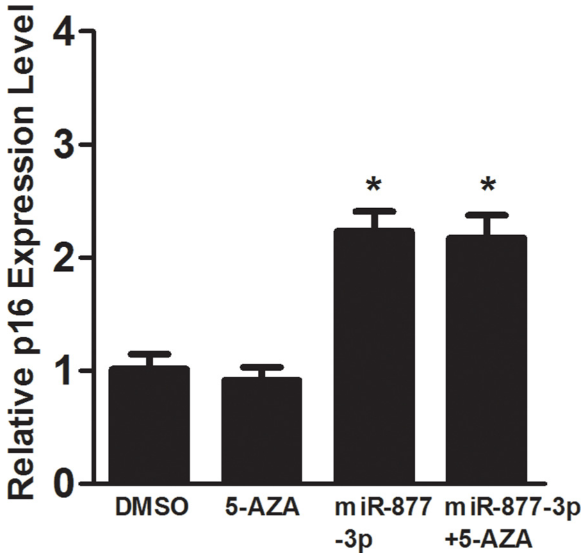

Supplementary Figure S1: p16 expression pattern after treaement with 5-Aza. DNA methylation inhibitor 5-Aza could not change the expression pattern of p16 in T24 cells. T24 cells were treated with DMSO, 5-Aza, miR-877-3p or co-treated with 5-AZA and miR-877-3p. 72 h after treatment, real-time PCR was performed to detect the expression of p16. GAPDH was used as inter control. 5-AZA did not affect the expression pattern of p16 in T24 cells (*$P < 0.05$).

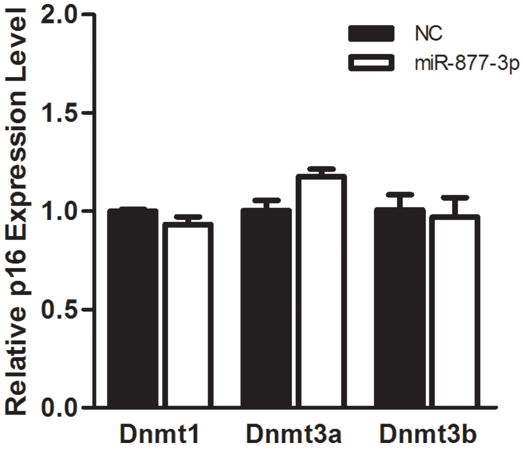

Supplementary Figure S2: Expressions of DNA methyltrans-ferases after transfection with miR-877-3p. Expressions of DNA methyltrans-ferases in T24 cells. After transfection with miR-877-3p for 72 h in T24 cells, real-time PCR was used to analyze the expression of DNA methyltrans-ferases, Dnmt1, Dnmt3a and Dnmt3b. GAPDH was severed as inter control. The expressions of Dnmt1, Dnmt3a and Dnmt3b showed no significant change.

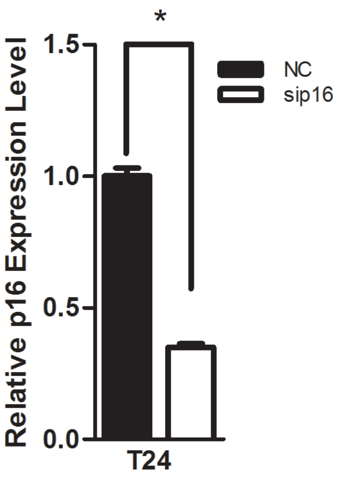

Supplementary Figure S3: Knock down efficiency of p16 siRNA. Knock down efficiency of p16 siRNA in T24 cells. Real-time PCR was performed to detect the expression of p16 after transfected with p16 siRNA for 72 h. GAPDH was regarded as inter control. The p16 expression level was reduced to 35% compared with the negative control (*$P < 0.05$).
